# Human population growth and the demographic transition

**DOI:** 10.1098/rstb.2009.0137

**Published:** 2009-10-27

**Authors:** John Bongaarts

**Affiliations:** The Population Council, One Dag Hammarskjold Plaza, New York, NY 10017, USA

**Keywords:** population growth, demographic transition, fertility, mortality, age structure

## Abstract

The world and most regions and countries are experiencing unprecedentedly rapid demographic change. The most obvious example of this change is the huge expansion of human numbers: four billion have been added since 1950. Projections for the next half century expect a highly divergent world, with stagnation or potential decline in parts of the developed world and continued rapid growth in the least developed regions. Other demographic processes are also undergoing extraordinary change: women's fertility has dropped rapidly and life expectancy has risen to new highs. Past trends in fertility and mortality have led to very young populations in high fertility countries in the developing world and to increasingly older populations in the developed world. Contemporary societies are now at very different stages of their demographic transitions. This paper summarizes key trends in population size, fertility and mortality, and age structures during these transitions. The focus is on the century from 1950 to 2050, which covers the period of most rapid global demographic transformation.

## 1. Introduction

After centuries of very slow and uneven growth, the world population reached one billion in 1800. The modern expansion of human numbers started then, rising at a slow but more steady pace over the next 150 years to 2.5 billion in 1950. During the second half of the twentieth century, however, growth rates accelerated to historically unprecedented levels. As a result, world population more than doubled to 6.5 billion in 2005 (United Nations 1962, 1973, 2007). This population expansion is expected to continue for several more decades before peaking near 10 billion later in the twenty-first century. Around 2070, the world's population will be 10 times larger than in 1800.

The recent period of very rapid demographic change in most countries around the world is characteristic of the central phases of a secular process called the *demographic transition*. Over the course of this transition, declines in birth rates followed by declines in death rates bring about an era of rapid population growth. This transition usually accompanies the development process that transforms an agricultural society into an industrial one. Before the transition's onset, population growth (which equals the difference between the birth and death rate in the absence of migration) is near zero as high death rates more or less offset the high birth rates typical of agrarian societies before the industrial revolution. Population growth is again near zero after the completion of the transition as birth and death rates both reach low levels in the most developed societies. During the intervening transition period, rapid demographic change occurs, characterized by two distinct phases. During the first phase, the population growth rate rises as the death rate declines while the birth rate remains high. In the second phase, the growth rate declines (but remains positive) due to a decline in the birth rate. The entire transition typically takes more than a century to complete and ends with a much larger population size.

**Figure 1.**
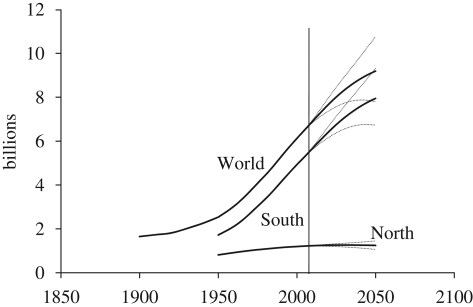
Population size estimates, 1900–2005 and projections 2005–2050. High, medium and low variants.

The plot of world population size over time in figure 1 (top solid line) shows the typical S-shaped pattern of estimated and projected population size over the course of the transition. Population growth accelerated for most of the twentieth century reaching the transition's midpoint in the 1980s and has recently begun to decelerate slightly. Today, we are still on the steepest part of this growth curve with additions to world population exceeding 75 million per year between 1971 and 2016.

Contemporary societies are at very different stages of their demographic transitions. Key trends in population size, fertility and mortality during these transitions are summarized below. The focus is on the century from 1950 to 2050, covering the period of most rapid global demographic change. The main source of data is the United Nation's 2006 world population assessment, which provides estimates for 1950–2005 and projections from 2005 to 2050 (United Nations 2007).

## 2. Future population trends

The projected rise in world population to 9.2 billion in 2050 represents an increase of 2.7 billion over the 2005 population of 6.5 billion. Nearly all of this future growth will occur in the ‘South’—i.e. Africa, Asia (excluding Japan, Australia and New Zealand), and Latin America—where population size is projected to increase from 5.3 to 7.9 billion between 2005 and 2050 (table 1). In contrast, in the ‘North’ (Europe, Northern America, Japan and Australia/New Zealand), population size is forecast to remain virtually stable, growing slightly from 1.22 to 1.25 billion between 2005 and 2050. The difference in trends between these two world regions reflects the later stage of the transition in the North compared with the South.

**Table 1.** Population estimates (1950–2005) and projections (2005–2050), by region. Adapted from United Nations (2007).

	population (billions)			% increase	
	1950	2005	2050	1950–2005	2005–2050
Africa	0.22	0.92	2.00	311	117
Sub-Saharan	0.18	0.77	1.76	327	129
Asia	1.41	3.94	5.27	179	34
Latin America	0.17	0.56	0.77	233	38
Europe	0.55	0.73	0.66	33	−9
Northern America	0.17	0.33	0.45	94	34
South	1.72	5.30	7.95	208	50
North	0.81	1.22	1.25	49	2
World	2.54	6.51	9.19	157	41

The global demographic transition began in the nineteenth century in the now economically developed parts of the world (the North) with declines in death rates. Large reductions in birth rates followed in the early part of the twentieth century. These transitions are now more or less complete. But, as shown in table 1, trends for the two principal regions in the North are expected to diverge between 2005 and 2050: an increase from 0.33 to 0.45 billion in Northern America, and a decline from 0.73 to 0.66 billion in Europe. In fact, several countries in Europe (e.g. Russia) and East Asia (e.g. Japan) face significant population declines as birth rates have fallen below death rates.

The demographic transitions in Africa, Asia and Latin America started later and are still underway. In 2005, Asia had a population of 3.94 billion, more than half of the world total, and its population is expected to grow by 34 per cent to 5.27 billion by 2050. Africa, with 0.92 billion inhabitants in 2005, is likely to experience by far the most rapid relative expansion, more than doubling to 2.0 billion by 2050. Latin America, with 0.56 billion in 2005, is the smallest of the regions of the South; its projected growth trend is similar to that of Asia.

It may seem surprising that population growth continues at a rapid pace in sub-Saharan Africa, where the AIDS epidemic is most severe. This epidemic has indeed caused many deaths, but population growth continues because the epidemic is no longer expanding and the birth rate is expected to remain higher than the elevated death rate in the future (UNAIDS 2007; Bongaarts *et al*. 2008). The epidemic's demographic impact can be assessed by comparing the standard UN population projection (which includes the epidemic's effect) with a separate hypothetical projection in which AIDS mortality is excluded (United Nations 2007). In sub-Saharan Africa, the former projects a 2050 population of 1.76 billion and the latter a population of 1.95 billion. The difference of 0.2 billion in 2050 between these projections with and without the epidemic is due to deaths from AIDS as well as the absence of the descendents from people who died from AIDS. According to these projections, the population of sub-Saharan Africa will grow by one billion between 2005 and 2050 despite the substantial impact of the AIDS epidemic. In fact, no country is expected to see a decline in its population size between 2005 and 2050 due to high AIDS mortality. Most populations in sub-Saharan Africa will more than double in size, several will triple and Niger is expected to quadruple by 2050 (United Nations 2007).

Transitions in the developing world have generally produced more rapid population growth rates in mid-transition than historically observed in the North. In some developing countries (e.g. Kenya and Uganda), peak growth rates approached four per cent per year in recent decades (implying a doubling of population size in two decades), levels that were very rarely observed in developed countries except with massive immigration. Two factors account for this very rapid expansion of population in these still largely traditional societies: the spread of medical technology (e.g. immunization, antibiotics) after World War II, which led to extremely rapid declines in death rates, and a lag in declines in birth rates.

Population sizes for the 10 largest countries in 2005 and in 2050 are presented in table 2. In 2005, China (1.31 billion) and India (1.13 billion) were by far the largest countries, together accounting for nearly half the South's total. The top 10 include six Asian countries and only one country each in Latin America and Africa. By 2050, the ranking is expected to have shifted substantially, with India's population exceeding China's, and with Ethiopia and DR Congo rising to the top 10, replacing Japan and the Russian Federation.

**Table 2.** Ten largest countries by population size in 1995 (estimate) and 2050 (medium projection). Adapted from United Nations (2007).

rank	1995		2050	
	country	population size (millions)	country	projected population (millions)
1	China	1313	India	165
2	India	1134	China	140
3	United States	300	United States	40
4	Indonesia	226	Indonesia	29
5	Brazil	187	Pakistan	29
6	Pakistan	158	Nigeria	28
7	Bangladesh	153	Brazil	25
8	Russian Federation	144	Bangladesh	25
9	Nigeria	141	D.R. Congo	18
10	Japan	128	Ethiopia	18

To simplify the presentation of results, all projections discussed in this study are taken from the medium variant of the UN projections (United Nations 2007). The UN has a good record of making relatively accurate projections (National Research Council 2000), but the future is of course uncertain and actual population trends over the next half century will likely diverge to some extent from current projections. The UN makes an effort to capture this uncertainty by publishing separate high and low projections. For the world, the high and low variants reach 7.8 and 10.8 billion, respectively, in 2050, indicating a rather wide range of possible outcomes (see dashed lines in figure 1).

## 3. Drivers of population growth: fertility and mortality

The world's population increases every year because the global birth rate exceeds the death rate. For example, in 2000–2005 population size increased at a rate of 1.17 per cent per year, which equals the difference between a birth rate of 2.03 per cent and a death rate of 0.86 per cent. At the country level, population growth is also affected by migration, but for the regional aggregates of population used in this analysis, migration is usually a minor factor, and it will therefore not be discussed in detail.

The annual birth and death rates of populations are in turn primarily determined by levels of fertility and mortality experienced by individuals. The most widely used fertility indicator is the total fertility rate (TFR), which equals the number of births a woman would have by the end of her reproductive years if she experienced the age-specific fertility rates prevailing in a given year. Mortality is often measured by the life expectancy (LE) at birth, which equals the average number of years a newborn would live if subjected to age-specific mortality rates observed in a given year.

### (a) Fertility

**Figure 2.**
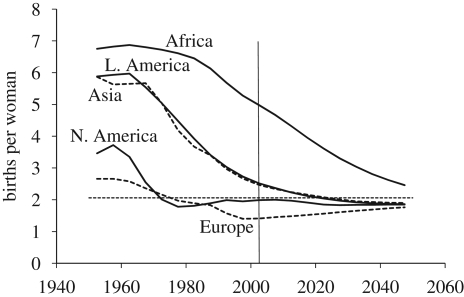
Trends in the total fertility rate by region.

The UN's past estimates and future projections of fertility levels by region for the period 1950–2050 are presented in figure 2. In the 1950s, the TFR in the South was high and virtually stable at around six births per woman on average. This high level of fertility reflects a near absence of birth control, a condition that has prevailed for centuries before the middle of the twentieth century. In the late 1960s, a rapid decline in fertility started nearly simultaneously in Asia and Latin America. In contrast, Africa has experienced only limited reproductive change. As a result of these divergent past trends, fertility levels in 2000–2005 differed widely among regions from as high as 5 births per woman (bpw) in Africa, to 2.5 bpw in Asia and Latin America. Average fertility in the North was already low in the early 1950s and has since declined to 2.0 bpw in Northern America and to 1.4 bpw in Europe.

The decline in the average fertility in the South from 6 to 3 bpw over the past half century has been very rapid by historical standards. This reproductive revolution is mainly due to two factors. First, desired family size of parents has declined as the cost of children rose and child survival increased. Second, government intervention played a key role. In China this took the form of a coercive and unpopular one-child policy, but most other countries implemented voluntary family planning programmes. The aim of these programmes is to provide information about and access to contraceptives at subsidized prices so that women who want to limit their childbearing can more readily do so.

UN projections for the South assume that the TFR will eventually reach and then fall slightly below the so-called ‘replacement’ level in all regions. Replacement fertility is just above 2 bpw and it represents the level at which each generation just replaces the previous one, thus leading to zero population growth (in the absence of mortality change and migration). Below-replacement fertility produces, in the long run, population decline. As is evident from figure 2, the TFRs in Asia and Latin America are expected to reach the replacement level around 2020. Africa is assumed to be on a much slower trajectory towards replacement fertility because of its lower level of socio-economic development. High fertility therefore remains a key cause of future population growth in this region. In contrast, the already low fertility of the North is expected to remain below replacement and is no longer driving population growth.

### (b) Mortality and life expectancy

**Figure 3.**
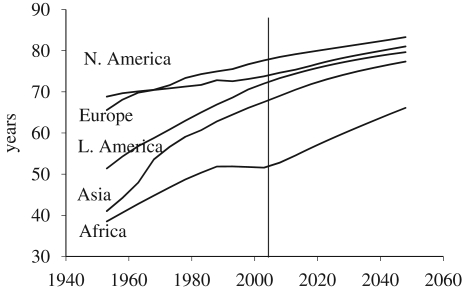
Trends in LE by region.

Mortality levels have also changed rapidly over the past several decades (figure 3). The South experienced exceptional improvements in LE from an average of 41 years in 1950–1955 to 64 years in 2000–2005. By the early 2000, Latin America reached mortality levels similar to those prevailing in the North in the 1970s, and Asia was just a few years behind. Africa experienced the highest mortality and improvements in LE stalled in the 1990s due to the AIDS epidemic. As a result, Africa's LE, at 52 years in 2000–2005, was still substantially below that of Asia (68) and Latin America (72). As expected, Europe and Northern America already achieved relatively low levels of mortality by 1950, but they have nevertheless seen significant further improvements since then. Europe's LE (74) is now lower than North America's (78) because of a rise in mortality in Eastern Europe after the break-up of the Soviet Union.

Projections of future LEs by the UN assume continued improvements over time in all regions. The North is expected to reach 82 years in 2050 despite the increasing difficulty in achieving increments as countries reach ever higher levels of LE. Asia and Latin America are expected to continue to close the gap with the North, and Africa will continue to lag, in part because the continent remains affected by the AIDS epidemic.

It should be noted that the assumptions made by the UN about future trends in fertility and mortality are not based on a firm theoretical basis. Instead, the UN relies on empirical regularities in past trends in countries that have completed their transitions, mostly in the North, where fertility declined to approximately the replacement level, and increases in LE became smaller over time. This is a plausible approach that unfortunately leaves room for potential inaccuracies in projection results.

## 4. Changing population age composition

Over the course of the demographic transition, declines in fertility and mortality cause important changes in a population's age composition. In general, countries in the early stages of the transition have a younger age structure than countries in the later stages.

**Figure 4.**
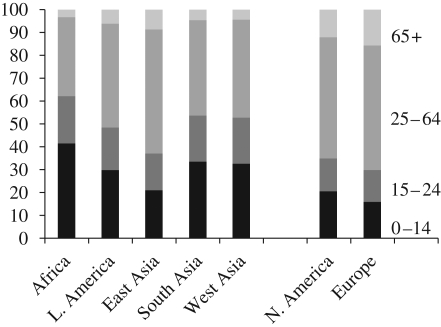
Distribution of population by age, by region, 2005.

Figure 4 presents the distribution of the 2005 population in four broad age groups: 0–14, 15–24, 25–64 and 65+ by region. Most of the regions in the South—Africa, Latin America, South Asia and West Asia—have very young age structures with about half of the population under age 25 (62% in Africa). The exception is East Asia (mostly China) where this proportion is 37 per cent. In the North, the population under 25 is still smaller: 35 per cent in North America and just 30 per cent in Europe. The reverse pattern is observed for the proportion 65+, which is much higher in the North than in the South, ranging from as high as 15 per cent in Europe to as low as just 3 per cent in Africa.

### (a) The age-dependency ratio

A changing age distribution has significant social and economic consequences, e.g. for the allocation of education, healthcare and social security resources to the young and old. Assessments of this impact often rely on the so-called age-dependency ratio (DR) that summarizes key changes in the age structure. The DR at a given point in time equals the ratio of population aged below 15 and over 65 to the population of age 15–64. This ratio aims to measure how many ‘dependents’ there are for each person in the ‘productive’ age group. Obviously, not every person below 15 and over 65 is a dependent and not every person between ages 15 and 65 is productive. Despite its crudeness, this indicator is widely used to document broad trends in the age composition.

**Figure 5.**
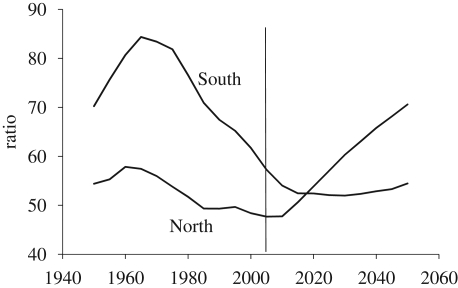
Dependency ratio estimates, 1950–2005.

Over the course of a demographic transition, the DR shows a characteristic pattern of change. Figure 5 presents this pattern as observed in the South from 1950 to 2005 and projected from 2005 to 2050. Early in the transition, the DR typically first rises slightly as improvements in survival chances of children raise the number of young people. Next, the DR falls sharply as declines in fertility reduce the proportion of the population under age 15. This decline has important economic consequences because it creates a so-called ‘demographic dividend’, which boosts economic growth by increasing the size of the labour force relative to dependents and by stimulating savings (Birdsall *et al*. 2001). Finally, at the end of the transition, the DR increases again as the proportion of the population over age 65 rises. Figure 5 also plots the DR of the North from 1950–2050. From 1950 to 2010 it showed a slight decline, but after 2010 it rises steeply as very low fertility and increasing longevity increases the proportion 65+. This ageing of the North poses serious challenges to support systems for the elderly (OECD 1998, 2001).

### (b) Population momentum

At the end of the demographic transition natural population growth reaches zero once three conditions are met:

- Fertility levels-off at the replacement level of about 2.1 bpw (more precisely, the net reproduction rate should be 1). If fertility remains above replacement, population growth continues.
- Mortality stops declining. In practice, this is not likely to happen because improvements in medical technology and healthcare as well as changes in lifestyles, etc. will probably ensure continued increases in LE.
- The age structure has adjusted to the post-transitional levels of fertility and mortality.

The adjustment of the age structure at the end of the transition takes many decades to complete. A key implication of this slow adjustment process is that population growth continues for many years after replacement fertility is reached if, as is often the case, the population is still relatively young when fertility reaches the replacement level. The tendency of population size to increase after a two-child family size has been reached is referred to as *population momentum*; it is the consequence of a young population age structure (‘young’ is defined relative to the age structure in the current life table) (Bongaarts & Bulatao 1999).

The population momentum inherent in the age structure of a particular population at a given point in time can be estimated with a hypothetical population projection in which future fertility is set instantly to the replacement level, mortality is held constant and migration is set to zero. Since such a variant is not directly available from UN projections, it will not be presented here. However, the UN does provide ‘instant replacement’ projections in which mortality and migration trends are the same as in the standard projection. This projection gives an approximation of the combined effect on future growth of population momentum and declining mortality in the South because the role of migration is small. The difference between this hypothetical projection and the standard medium UN projection is a measure of the impact of high fertility on future population growth.

**Figure 6.**
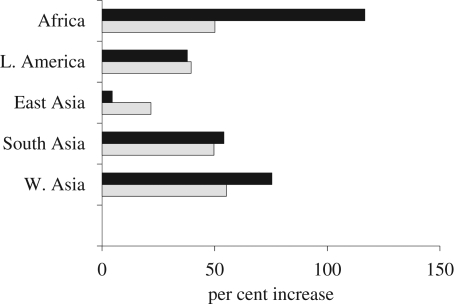
Percentage increase in population 2005–2050, by region, alternative projections. Black bars denote medium UN projection; grey bars denote instant replacement projection (hypothetical).

Results of these two projections are presented in figure 6, which compares the per cent growth between 2005 and 2050 for regions in the South. The black bars give the growth in the standard (medium variant) projection and the grey bars give the growth in the ‘instant replacement’ projection. Three results are noteworthy. First, the two projections differ most in Africa (+117% versus +50%) which is as expected because fertility is still very high in this region. Second, in all regions of the South outside China, populations would be expected to rise by 50 per cent (62% in West Asia) if fertility were set to replacement in 2005. This implies that momentum and declining mortality are responsible for nearly half of the projected future population growth in Africa and for the large majority of growth in Latin America, and South and West Asia. Third, in East Asia and in Latin America the replacement projection exceeds the medium UN projection. This finding is explained by the fact that fertility in these regions is assumed to average below the replacement level over the next half century.

## 5. Conclusion

The world and most countries are going through a period of unprecedentedly rapid demographic change. The most obvious example of this change is the huge expansion of human numbers: four billion have been added since 1950. Other demographic processes are also experiencing extraordinary change: women are having fewer births and LEs have risen to new highs. Past trends in fertility and/or mortality have led to very young populations in high fertility countries in the South and to increasingly older populations in the North. Still other important demographic changes which were not reviewed here include rapid urbanization, international migration, and changes in family and household structure.

Global population growth will continue for decades, reaching around 9.2 billion in 2050 and peaking still higher later in the century. The demographic drivers of this growth are high fertility in parts of the South, as well as declining mortality and momentum. This large expansion in human numbers and of the accompanying changes in the age structure will have multiple consequences for society, the economy and the environment as discussed in the subsequent chapters in this issue.
